# Increased risk for diabetes mellitus in patients with carbon monoxide poisoning

**DOI:** 10.18632/oncotarget.18887

**Published:** 2017-06-29

**Authors:** Chien-Cheng Huang, Chung-Han Ho, Yi-Chen Chen, Hung-Jung Lin, Chien-Chin Hsu, Jhi-Joung Wang, Shih-Bin Su, How-Ran Guo

**Affiliations:** ^1^ Department of Emergency Medicine, Chi Mei Medical Center, Tainan, Taiwan; ^2^ Department of Environmental and Occupational Health, College of Medicine, National Cheng Kung University, Tainan, Taiwan; ^3^ Bachelor Program of Senior Service, Southern Taiwan University of Science and Technology, Tainan, Taiwan; ^4^ Department of Geriatrics and Gerontology, Chi Mei Medical Center, Tainan, Taiwan; ^5^ Department of Occupational Medicine, Chi Mei Medical Center, Tainan, Taiwan; ^6^ Department of Medical Research, Chi Mei Medical Center, Tainan, Taiwan; ^7^ Department of Pharmacy, Chia Nan University of Pharmacy and Science, Tainan, Taiwan; ^8^ Department of Biotechnology, Southern Taiwan University of Science and Technology, Tainan, Taiwan; ^9^ Department of Emergency Medicine, Taipei Medical University, Taipei, Taiwan; ^10^ Department of Leisure, Recreation and Tourism Management, Southern Taiwan University of Science and Technology, Tainan, Taiwan; ^11^ Department of Medical Research, Chi Mei Medical Center, Tainan, Taiwan; ^12^ Department of Occupational and Environmental Medicine, National Cheng Kung University Hospital, Tainan, Taiwan

**Keywords:** brain, carbon monoxide, endocrine, diabetes mellitus, poisoning

## Abstract

Carbon monoxide poisoning (COP) causes hypoxic injury and inflammatory and immunological reactions in the brain and local organs including the pancreas. Therefore, it is plausible that COP may increase the risk for developing diabetes mellitus (DM), but studies on this possible association are limited. We conducted a nationwide study in Taiwan to fill the data gap. We used the Nationwide Poisoning Database and the Longitudinal Health Insurance Database 2000 to identify all COP patients diagnosed between 1999 and 2012 (the study cohort) and then construct a comparison cohort of patients without COP through matching at 1:3 by the index date and age. The risk for DM between the two cohorts was compared by following up until 2013. We also investigated the independent predictors for DM in all the patients. During the study period, 22,308 COP patients were identified, and 66,924 non-COP patients were included in the comparison cohort accordingly. Patients with COP had an increased risk for DM with an adjusted hazard ratio (AHR) of 1.92 (95% confidence interval [CI]: 1.79–2.06) after adjusting for age, sex, comorbidities, and monthly income, especially in the subgroups of age <35 years, age ≥ 65 years, female sex, and comorbidities with congestive heart failure, hyperthyroidism, and polycystic ovary syndrome. Cox proportional hazard regression analysis showed that the increased risk for DM was highest in the first month after COP (AHR= 3.38; 95% CI: 2.29–4.99) and lasted even after 4 years (AHR= 1.82; 95% CI: 1.62–2.04). We found that COP, older age, male sex, hypertension, hyperlipidemia, hyperuricemia, and low monthly income were independent predictors for DM. Intervention studies are needed to validate the results and delineate the detailed mechanisms.

## INTRODUCTION

Carbon monoxide (CO) is produced from an incomplete combustion of organic compounds [1]. Because it has a 250 times greater affinity for hemoglobin than for oxygen, even a small degree of CO poisoning (COP) could lead to severe hypoxia [2]. The main causes of unintentional COP are inadequately ventilated gas from heating appliances, fires, and automobile exhaust fumes [1]. Increasing numbers of intentional COP cases due to charcoal burning have been reported, because CO is odorless but fatal, which is believed to be a perfect tool for suicide [3]. In Taiwan, a 25-fold increase of suicidal COP by charcoal burning has been reported, from 0.22 in 1999 to 5.4/100,000 people in 2009, which contributed remarkably to an increased national suicide rate during this period from 10.4 to 19.3 for every 100,000 people [3].

The most common morbidity after COP is neurological sequelae, because the brain is one of the most vulnerable organs to hypoxia [4–8]. In addition to hypoxia, COP may induce immunological and inflammatory reactions in all organs in the human body by producing reactive oxygen species, which are longer lasting and independent of hypoxia [4, 5].

The brainstem and the hypothalamus have glucose-sensing or glucose-inhibiting neurons, which can control the autonomic nervous system and affect the metabolic state of the liver, muscles, and fat tissue and the secretory activity of the pancreas [9]. Therefore, COP-induced brain and local organ (especially pancreas) injuries may affect the glucose homeostasis and even result in the development of diabetes mellitus (DM). However, we did not find any study on the association between COP and the risk for DM upon searching the PubMed and Google Scholar using the keywords “carbon monoxide,” “poisoning,” “diabetes mellitus,” “brain,” and “endocrine.” Therefore, we conceived this study to clarify this issue, which remains unclear.

## RESULTS

The mean age of patients in both cohorts was 34.9 (standard deviation= 14.5) years (Table 1). The age subgroup with the largest number of members in both cohorts by was 20–34 years (41.87%), followed by 35–49 years (31.96%). There were more women than men in both cohorts (*p* = 0.023). Patients with COP had a higher prevalence of comorbidities of hypertension, hyperlipidemia, hyperuricemia, coronary artery disease, congestive heart failure, and gestational DM, but had a lower monthly income than non-COP patients.

**Table 1:** Comparison of age, sex, comorbidities, and monthly income between both cohorts

Variable	COP cohort(n = 22,308)	Comparison cohort(n = 66,924)	*p*-value
Age (year)	34.9 ± 14.5	34.9 ± 14.5	0.990
Age (year)			
<20	2682 (12.02)	8049 (12.02)	>0.999
20–34	9340 (41.87)	28018 (41.87)	
35–49	7130 (31.96)	21390 (31.96)	
50–64	2312 (10.36)	6935 (10.36)	
≥65	844 (3.78)	2532 (3.78)	
Sex			
Female	11398 (51.09)	34784 (51.98)	0.023
Male	10910 (48.91)	32140 (48.02)	
Comorbidity			
Hypertension	1739 (7.80)	4554 (6.80)	<0.001
Hyperlipidemia	977 (4.38)	2559 (3.82)	<0.001
Hyperuricemia	880 (3.94)	2430 (3.63)	0.032
Obstructive sleep apnea	–	–	
Coronary artery disease	812 (3.64)	1653 (2.47)	<0.001
Congestive heart failure	216 (0.97)	322 (0.48)	<0.001
Hyperthyroidism	326 (1.46)	863 (1.29)	0.053
Hypothyroidism	88 (0.39)	230 (0.34)	0.270
Gestational DM	5 (0.02)	48 (0.07)	0.009
Polycystic ovary syndrome	99 (0.44)	280 (0.42)	0.613
Monthly income (NTD)			
<19,999	15961 (71.55)	41048 (61.34)	<0.001
20,000–39,999	5077 (22.76)	19151 (28.62)	
≥40,000	1270 (5.69)	6725 (10.05)	

COP, carbon monoxide poisoning; DM, diabetes mellitus; NTD, New Taiwan dollars. Data are expressed as mean ± standard deviation or n (%).

**Figure 1:**
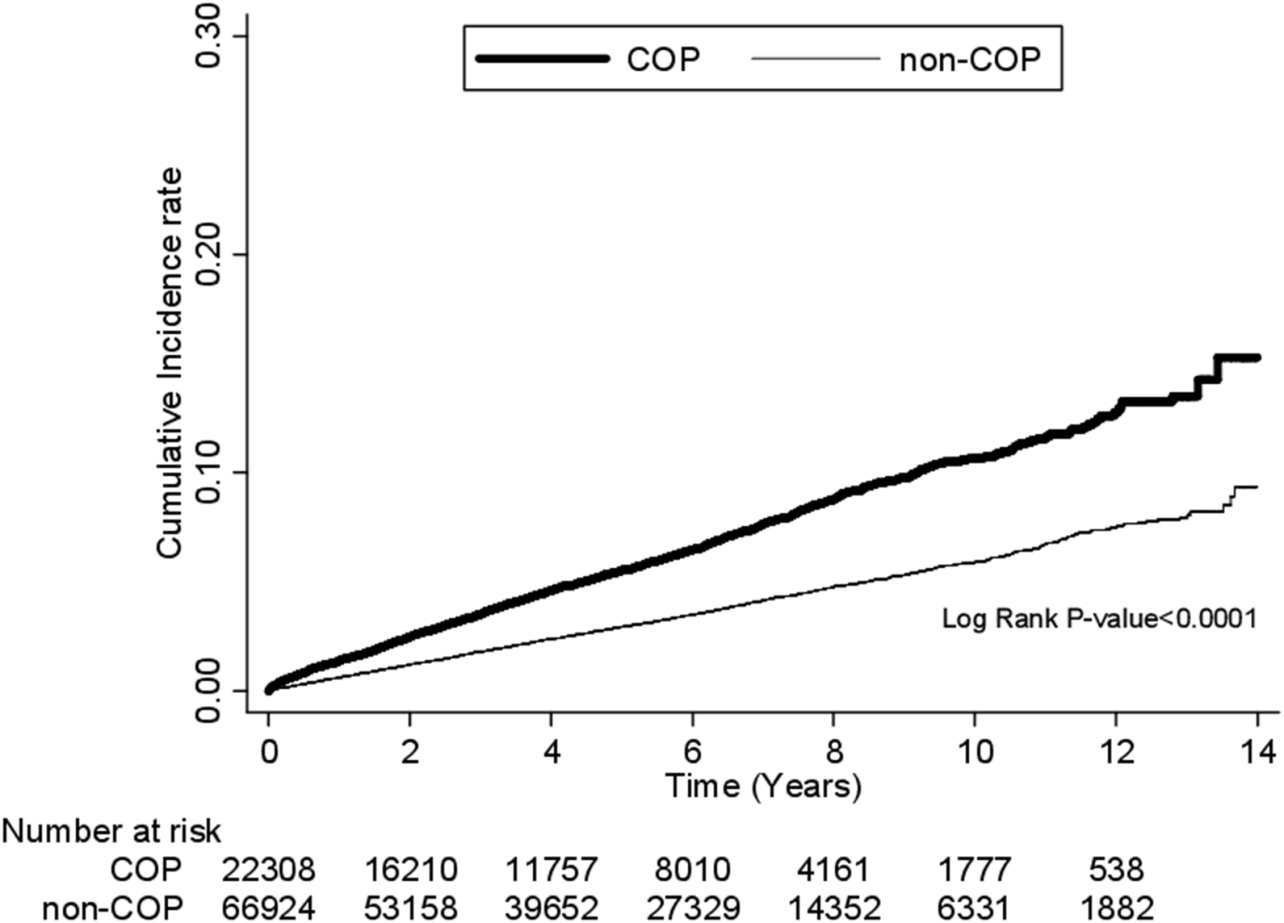
Comparison of the risk for DM between COP and non-COP patients by Kaplan-Meier's method and log-rank test DM, diabetes mellitus; COP, carbon monoxide poisoning.

During the follow-up, patients with COP had a higher risk for DM than non-COP patients, with an adjusted hazard ratio (AHR) of 1.92 (95% confidence interval [CI]: 1.79–2.06). The subgroups with an AHR above the overall value of 1.92 included age <20 years (AHR= 2.36; 95% CI: 1.28–4.35), 20–34 years (AHR= 2.67; 95% CI: 2.26–3.15) and ≥ 65 years (AHR= 2.22; 95% CI: 1.82–2.72), female sex (AHR= 2.35; 95% CI: 2.12–2.60), and comorbidities of congestive heart failure (AHR= 2.64; 95% CI: 1.64–4.24), hyperthyroidism (AHR= 2.86; 95% CI: 1.58–5.16), and polycystic ovary syndrome (AHR= 4.88; 95% CI: 1.53–15.6) (Table 2). While the AHR (2.43) in patients with hypothyroidism was also above 1.92, it did not reach statistical significance (95% CI: 0.66–8.97). The increased risk for DM in patients with COP was highest in the first month after COP (AHR= 3.38; 95% CI: 2.29–4.99) and persisted even after 4 years of follow-up (AHR= 1.82; 95% CI: 1.62–2.04). Kaplan-Meier's method and log-rank test also showed an increased risk for DM in patients with COP compared to that in non-COP patients (*p* < 0.001) (Figure 1).

**Table 2:** Comparison of the risk for DM between the two cohorts using Cox proportional hazard regression analysis

Variable	COP cohort			Comparison cohort			Crude HR(95% CI)	AHR*(95% CI)	*p*-value†
	Case (%)	PY	Rate	Case (%)	PY	Rate			
Overall analysis	1236 (5.54)	106754.63	11.58	2158 (3.22)	354187.43	6.09	1.90 (1.77–2.04)	1.92 (1.79–2.06)	<0.001
Stratified analysis									
Age (year)									
<20	18 (0.67)	16725.31	1.08	25 (0.31)	51503.50	0.49	2.22 (1.21–4.07)	2.36 (1.28–4.35)	0.006
20–34	257 (2.75)	46155.73	5.57	310 (1.11)	149751.10	2.07	2.70 (2.29–3.19)	2.67 (2.26–3.15)	<0.001
35–49	517 (7.25)	32660.74	15.83	966 (4.52)	111153.60	8.69	1.83 (1.64–2.03)	1.75 (1.57–1.95)	<0.001
50–64	290 (12.54)	8452.96	34.31	589 (8.49)	30583.48	19.26	1.78 (1.55–2.05)	1.70 (1.48–1.96)	<0.001
≥65	154 (18.25)	2759.89	55.8	268 (10.58)	11195.75	23.94	2.28 (1.87–2.79)	2.22 (1.82–2.72)	<0.001
Sex									
Female	629 (5.52)	56121.77	11.21	954 (2.74)	185759.9	5.14	2.18 (1.97–2.41)	2.35 (2.12–2.60)	<0.001
Male	607 (5.56)	50632.86	11.99	1204 (3.75)	168427.5	7.15	1.68 (1.52–1.85)	1.63 (1.48–1.80)	<0.001
Comorbidity									
Hypertension	269 (15.47)	5748.45	46.8	541 (11.88)	18452.4	29.32	1.59 (1.37–1.84)	1.56 (1.34–1.81)	<0.001
Hyperlipidemia	128 (13.10)	3236.59	39.55	287 (11.22)	9258.35	31.00	1.28 (1.04–1.58)	1.31 (1.06–1.62)	0.013
Hyperuricemia	117 (13.30)	3214.89	36.39	256 (10.53)	10234.58	25.01	1.46 (1.17–1.81)	1.44 (1.15–1.80)	0.001
Obstructive sleep apnea	–	–		–	–		–	–	
Coronary artery disease	122 (15.02)	2781.21	43.87	194 (11.74)	6507.96	29.81	1.47 (1.17–1.84)	1.57 (1.25–1.98)	<0.001
Congestive heart failure	40 (18.52)	563.61	70.97	32 (9.94)	1106.33	28.92	2.44 (1.53–3.88)	2.64 (1.64–4.24)	<0.001
Hyperthyroidism	22 (6.75)	1289.08	17.07	25 (2.9)	3773.82	6.62	2.59 (1.46–4.59)	2.86 (1.58–5.16)	<0.001
Hypothyroidism	4 (4.55)	366.63	10.91	12 (5.22)	954.96	12.57	0.85 (0.27–2.63)	2.43 (0.66–8.97)	0.182
Gestational DM	1 (20.00)	11.50	86.96	1 (2.08)	210.30	4.76	12.86 (0.79–208.07)	–	
Polycystic ovary syndrome	8 (8.08)	340.4	23.5	5 (1.79)	998.05	5.01	4.68 (1.53–14.3)	4.88 (1.53–15.6)	0.008
Follow-up period									
<1 month	57 (0.26)	1792.05	31.81	48 (0.07)	5549.47	8.65	3.67 (2.50–5.39)	3.38 (2.29–4.99)	<0.001
1–6 months	118 (0.55)	8600.47	13.72	153 (0.23)	27142.92	5.64	2.43 (1.91–3.09)	2.37 (1.86–3.02)	<0.001
7–12 months	109 (0.54)	9717.09	11.22	191 (0.30)	31059.69	6.15	1.82 (1.44–2.31)	1.80 (1.42–2.28)	<0.001
1–2 years	197 (1.06)	17622.55	11.18	342 (0.57)	57164.11	5.98	1.87 (1.57–2.23)	1.85 (1.55–2.21)	<0.001
2–4 years	308 (1.90)	28015.34	10.99	552 (1.04)	93060.43	5.93	1.85 (1.61–2.13)	1.86 (1.62–2.14)	<0.001
≥4 years	447 (3.80)	41007.13	10.9	872 (2.20)	140210.81	6.22	1.75 (1.56–1.96)	1.82 (1.62–2.04)	<0.001

COP, carbon monoxide poisoning; HR, hazard ratio; AHR, adjusted hazard ratio; CI, confidence interval; PY, person-year; DM, diabetes mellitus.

*Adjusted for age, sex, hypertension, hyperlipidemia, hyperuricemia, obstructive sleep apnea, coronary artery disease, congestive heart failure, hyperthyroidism, hypothyroidism, gestational DM, polycystic ovary syndrome, and monthly income for AHR.

Cox proportional hazard regression analysis showed that COP, older age, male sex, hypertension, hyperlipidemia, hyperuricemia, and polycystic ovary syndrome were independent predictors for DM in the full model (Table 3). In the reduced model, the independent predictors were COP, older age, male sex, hypertension, hyperlipidemia, hyperuricemia, and monthly income <19,999 NTD. The difference in the risk for DM between COP patients with and without acute respiratory failure (ARF) (AHR = 1.33; 95% CI: 0.69–2.56) did not reach statistical significance, but those who received HBOT had a higher risk (AHR= 1.81; 95% CI: 1.59–2.07). The Cox proportional hazard regression analysis after propensity score matching also showed a higher risk for DM in COP patients than non-COP patients (AHR= 1.89; 95% CI: 1.76–2.02) (Table 4).

**Table 3:** Independent predictors for DM in all patients of the two cohorts by Cox proportional hazard regression analysis

Variable	Uni-variate modelcrude HR (95% CI)	Full modelAHR (95% CI)*	Reduced modelAHR (95% CI)†
Cohort			
Comparison (Non-COP)	1 (reference)	1 (reference)	1 (reference)
Study (COP)			
All COP	1.90 (1.77–2.04)	1.92 (1.79–2.06)	1.92 (1.79–2.06)
COP with ARF	1.49 (0.78–2.87)	1.32 (0.69–2.55)	1.33 (0.69–2.56)
COP with HBOT	1.68 (1.48–1.91)	1.81 (1.59–2.06)	1.81 (1.59–2.07)
COP with ARF and HBOT‡	–	–	–
Age (year)			
<20	1 (reference)	1 (reference)	1 (reference)
20–34	4.70 (3.44–6.41)	4.93 (3.61–6.73)	4.98 (3.63–6.76)
35–49	16.80 (12.41–22.76)	16.48 (12.15–22.36)	16.49 (12.16–22.37)
50–64	36.95 (27.20–50.21)	29.50 (21.66–40.19)	29.43 (21.61–40.90)
≥65	49.36 (36.06–67.57)	28.74 (20.84–39.64)	28.70 (20.81–39.57)
Sex			
Female	1 (reference)	1 (reference)	1 (reference)
Male	1.26 (1.18–1.35)	1.20 (1.12–1.29)	1.20 (1.12–1.28)
Comorbidity			
Hypertension	5.73 (5.29–6.20)	1.89 (1.70–2.09)	1.88 (1.70–2.09)
Hyperlipidemia	5.05 (4.55–5.60)	1.54 (1.37–1.73)	1.54 (1.37–1.73)
Hyperuricemia	4.13 (3.71–4.60)	1.56 (1.38–1.75)	1.56 (1.38–1.75)
Obstructive sleep apnea	–	–	–
Coronary artery disease	5.01 (4.46–5.62)	1.13 (0.99–1.29)	1.13 (0.99–1.29)
Congestive heart failure	5.94 (4.70–7.50)	1.25 (0.98–1.60)	1.25 (0.98–1.60)
Hyperthyroidism	1.27 (0.95–1.69)	0.95 (0.71–1.28)	–
Hypothyroidism	1.65 (1.01–2.69)	0.90 (0.55–1.48)	–
Gestational DM	1.23 (0.31–4.91)	1.93 (0.48–7.72)	–
Polycystic ovary syndrome	1.32 (0.76–2.27)	2.29 (1.32–3.95)	–
Monthly income (NTD)			
<19,999	1.13 (1.00–1.28)	0.88 (0.81–0.96)	1.27 (1.12–1.44)
20,000–39,999	0.86 (0.74–0.98)	0.79 (0.69–0.89)	1.12 (0.97–1.28)
≥40,000	1 (reference)	1 (reference)	1 (reference)

COP, carbon monoxide poisoning; HR, hazard ratio; AHR, adjusted hazard ratio; CI, confidence interval; ARF, acute respiratory failure; HBOT, hyperbaric oxygen therapy; DM, diabetes mellitus; NTD, New Taiwan Dollar.

*Adjusted for age, sex, hypertension, hyperlipidemia, hyperuricemia, obstructive sleep apnea, coronary artery disease, congestive heart failure, hyperthyroidism, hypothyroidism, gestational DM, polycystic ovary syndrome, and monthly income. †Adjusted for age, sex, hypertension, hyperlipidemia, hyperuricemia, coronary artery disease, congestive heart failure, and hypothyroidism. ‡ No patient developed DM in the subgroup of COP with ARF and HBOT.

**Table 4:** Independent predictors for DM after propensity score matching by Cox proportional hazard regression analysis

Variable	All COPn (%)	Non-COPn (%)	*p*-value	AHR (95%CI)*	*p*-value†
Cohort					
All COP vs. Non-COP	22308	66924		1.89 (1.76–2.02)	<0.001
Age (years)			0.999		
<20	2682 (12.02)	8036 (12.01)		1 (reference)	–
20–34	9340 (41.87)	28011 (41.85)		5.03 (3.67–6.89)	<0.001
35–49	7130 (31.96)	21383 (31.95)		16.97 (12.47–23.07)	<0.001
50–64	2312 (10.36)	6958 (10.40)		30.27 (22.16–41.35)	<0.001
≥65	844 (3.78)	2536 (3.79)		30.57 (22.11–42.28)	<0.001
Sex					
Female	11398 (51.09)	34189 (51.09)	0.985	1 (reference)	–
Male	10910 (48.91)	32735 (48.91)		1.19 (1.11–1.27)	<0.001
Comorbidity					
Hypertension	1739 (7.80)	5249 (7.84)	0.818	1.87 (1.69–2.06)	<0.001
Hyperlipidemia	977 (4.38)	2962 (4.43)	0.771	1.36 (1.21–1.52)	<0.001
Hyperuricemia	880 (3.94)	2645 (3.95)	0.96	1.55 (1.38–1.73)	<0.001
Obstructive sleep apnea	0 (0.00)	0 (0.00)	–	–	–
Coronary artery disease	812 (3.64)	2437 (3.64)	0.992	1.11 (0.99–1.26)	0.083
Congestive heart failure	216 (0.97)	633 (0.95)	0.765	1.11 (0.90–1.36)	0.338
Hyperthyroidism	326 (1.46)	956 (1.43)	0.722	1.19 (0.93–1.53)	0.171
Hypothyroidism	88 (0.39)	244 (0.36)	0.526	0.89 (0.55–1.44)	0.634
Gestational DM	5 (0.02)	12 (0.02)	0.779	4.46 (0.63–31.68)	0.135
Polycystic ovary syndrome	99 (0.44)	297 (0.44)	> 0.999	3.21 (2.04–5.06)	<0.001
Monthly income (NTD)					
<19,999	15961 (71.55)	47905 (71.58)	0.992	1.16 (1.01–1.33)	0.039
20,000–39,999	5077 (22.76)	15223 (22.75)		0.93 (0.80–1.09)	0.379
≥40,000	1270 (5.69)	3796 (5.67)		1 (reference)	–

DM, diabetes mellitus; COP, carbon monoxide poisoning; AHR, adjusted hazard ratio; CI, confidence interval; DM, diabetes mellitus; NTD, New Taiwan Dollar.

*Adjusted for age, sex, hypertension, hyperlipidemia, hyperuricemia, obstructive sleep apnea, coronary artery disease, congestive heart failure, hyperthyroidism, hypothyroidism, gestational DM, polycystic ovary syndrome, and monthly income. †For AHR.

## DISCUSSION

This study showed that patients with COP had a higher risk for DM than non-COP patients. COP-induced hypoxic injury and inflammatory and immunological reactions in the brain and other organs, including the pancreas, were suggested to be the cause for the increased risk for DM. The brainstem, the hypothalamus, the mouth, and the hepatoportal vein area have glucose-sensing cells and are linked to each other by nervous connections [9]. Activation of the brainstem and the hypothalamus activates the autonomic nervous system that innervates pancreatic α- and β-cells [9]. In addition, these distributed glucose-sensing systems also control food intake and energy expenditure [10, 11]. Therefore, impaired glucose sensing by COP may result in overeating, reduced energy expenditure, impaired suppression of glucagon secretion, and reduced insulin secretion, which eventually lead to obesity and even type 2 DM [9, 11–14]. As the brain controls the endocrine system including the development of DM, treatment via the brain has been proposed as a novel method to cure DM [15]. A recent study reported that injection of fibroblast growth factor 1 into the brain of various mouse and rat models of type 2 DM resulted in an excellent response for long-lasting reduction of blood glucose [15].

We found that patients aged <20 years and 20–34 years and of female sex seemed to be more vulnerable for developing DM after COP. These findings are similar to those in a previous study which found patients aged <30 years and of female sex were more likely to have long-term mortality after COP [6]. The authors explained that because younger patients and female patients might have fewer comorbidities than their counterparts, COP became a major contributing factor for the increase in mortality [6].

We also found that patients with comorbidities of congestive heart failure, hyperthyroidism, and polycystic ovary syndrome seemed to be more vulnerable for DM after COP. There was no direct study about the impact of COP on the risk for DM in patients with these comorbidities. However, there were studies on the association between these comorbidities themselves and DM. Congestive heart failure and DM have a bidirectional relationship [16]. DM combined other risk factors including obesity, hypertension, and coronary heart disease leads to the development of congestive heart failure [16]. On the other hand, congestive heart failure increases the risk for new type 2 DM due to excessive neurohumoral response and decreased muscular perfusion [16]. Abnormalities of thyroid hormones may contribute to type 2 DM because both of them have an intersecting underlying mechanism [17]. The probable mechanism could be attributed to perturbed genetic expression of a constellation of genes along with physiological aberrations leading to impaired glucose utilization and disposal in muscles, overproduction of hepatic glucose output, and enhanced absorption of splanchnic glucose [17]. All these abovementioned factors contribute to insulin resistance and, eventually, the development of DM [17]. Polycystic ovary syndrome is caused due to elevated androgens in women and is related to insulin resistance and DM, which is a genetic disorder of insulin action [18, 19].

The increased risk for DM in patients with COP was highest in the first month, suggesting that the presence of acute abnormalities in glucose metabolism after COP. There is little direct evidence from the literature, however, to support this assumption. Hyperglycemia occurs in 30%–40% of patients with an acute ischemic stroke (another type of brain injury vs. COP), which is primarily caused by a stress response [20, 21]. The relationship between acute ischemic stroke and DM is bidirectional [20, 22]. DM increases the risk for acute ischemic stroke, and acute ischemic stroke worsens the control of DM [20]. A portion of patients with hyperglycemia after acute stroke have preexisting but unrecognized DM, which needs HbA1C to help in differential diagnosis [20]. The diagnosis of DM in this study was based on the treating physicians, and therefore the highest risk for DM in the first month was not a transient stress response. This issue needs further study to clarify the underlying mechanisms.

This study has the strength of using a nationwide database, and the novel finding of the association between COP and DM is supported by the identification and adjustment for other well-known independent predictors for DM, including older age, male sex, hypertension, hyperlipidemia, hyperuricemia, and low income. Nonetheless, it still has some limitations. First, there are no data on family history of DM, impaired glucose tolerance, body weight and lifestyle including diet, physical inactivity, and tobacco use, which are also risk factors for DM and thus potential confounders of this study. However, we had matched age in both cohorts and adjusted for various comorbidities including hypertension, hyperlipidemia, and hyperuricemia, which may serve as surrogates for some of these variables and therefore the potential confounding effect could be minimized. Furthermore, because most of these variables, such as family history of DM and impaired glucose tolerance, were unlikely to have strong associations with COP in our study population (i.e. have very different prevalence rates between the COP and the non-COP cohorts), they were not likely to introduce remarkable confounding effects to our study results. Second, HBOT is one of the most important interventions for COP, but we could not investigate its effect on the development of DM directly because we used non-COP patients, who were unlikely to receive HBOT, as the comparison cohort. Nonetheless, we found that the risk of developing DM in COP patients who received HBOT was smaller than but very close to that in all COP patients combined (AHR 1.81 [95% CI: 1.59–2.07] vs. 1.92 [95% CI: 1.79–2.06]). Accordingly, we can infer that HBOT by itself will not increase, if not decrease, the risk of developing DM even though COP patients who received HBOT still had a higher risk than non-COP patients in our study. Further studies that make comparisons between COP patients with and without HBOT are warranted. Third, we used surrogates to estimate the variable and might thus introduce misclassifications. For example, we defined ARF by ICD-9 518.81 or 518.84 or management codes 960, 9601, 9602, 9603, 9604, 9605, 9390, 9391, or 311, and this might underestimate the incidence of ARF. In addition to the fact that we believe this is the best approach to identify ARF in the database we believe this is the most accurate way to define these variables in the database, the approach was applied to both the study and the comparison cohorts, and therefore it is not likely to lead to remarkable biases in our results. Fourth, although this is a nationwide study, whether the results could be generalized to other nations needs further studies for validation, owing to the differences in culture, race, and disease management.

## MATERIALS AND METHODS

### Data source

We used the Nationwide Poisoning Database (NPD) and the Longitudinal Health Insurance Database 2000 (LHID2000), two sub-databases of the Taiwan National Health Insurance Research Database, for this retrospective nationwide population-based cohort study. Because Taiwan National Health Insurance program covers nearly 100% of Taiwan's population, the NPD represents all the poisonings including COP in Taiwan [23]. The LHID2000 contains the registration and claim data of 1,000,000 individuals randomly selected from the original National Health Insurance Research Database [23]. Large, computerized databases derived from this system by the National Health Insurance Administration (the former Bureau of National Health Insurance, BNHI), Ministry of Health and Welfare (the former Department of Health, DOH), Taiwan, and maintained by the National Health Research Institutes, Taiwan, are provided to scientists in Taiwan for research purposes [23].

### Definition of study cohort (COP patients) and comparison cohort (non-COP patients) and variables

**Figure 2:**
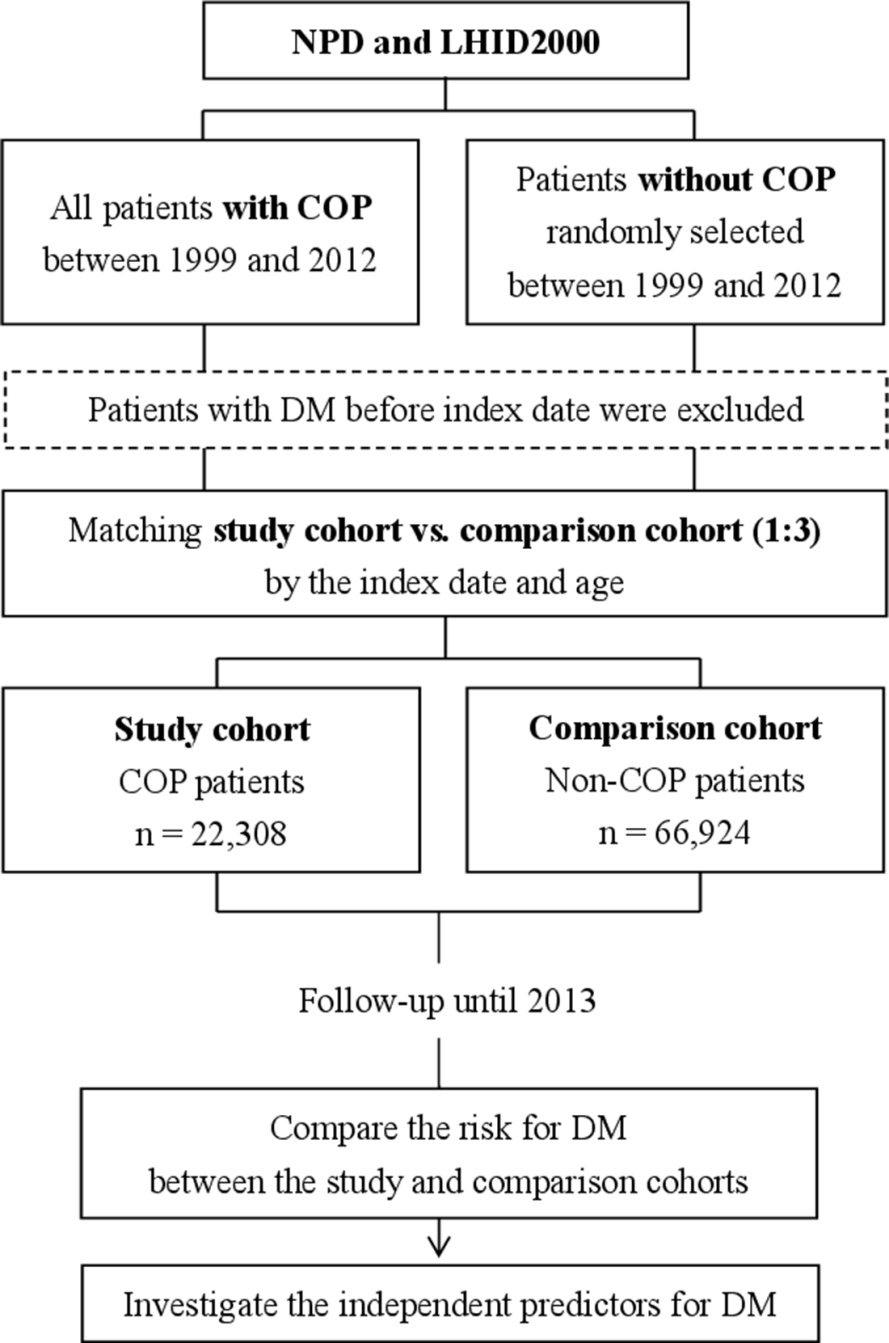
Flow chart of the study NPD, Nationwide Poisoning Database; LHID, Longitudinal Health Insurance Database; COP, carbon monoxide poisoning; DM, diabetes mellitus.

We identified the study cohort as patients with COP in the NPD who were diagnosed between 1999 and 2012 (Figure 2). The comparison cohort included non-COP patients who were identified from the LHID2000 by matching the index date and age with COP patients at a ratio of 1:3. The index date was defined as the date of admission or visiting the emergency department care for COP patients. The diagnosis of COP was identified by the International Classification of Diseases-9 (ICD-9) codes of 986, E868, E952, or E982 during either admission or visiting the emergency department care. Because DM was the outcome we intended to compare, participants who had DM before the index date were excluded. Age subgroups were classified as <20, 20–34, 35–49, 50–64, and ≥65 years. We also studied comorbidities of hypertension (ICD-9 401–405), hyperlipidemia (ICD-9 272), hyperuricemia (ICD-9 790.6, 274), obstructive sleep apnea (ICD-9 327.23), coronary artery disease (ICD-9 410–414), congestive heart failure (ICD-9 428), hyperthyroidism (ICD-9 242), hypothyroidism (ICD-9 244), gestational DM (ICD-9 6480), and polycystic ovary syndrome (ICD-9 256.4), which are risk factors for DM. Monthly income was classified as <19,999, 20,000–39,999, ≥ 40,000 New Taiwan Dollars (NTD) [24]. Hyperbaric oxygen therapy (HBOT) was defined by management codes 47054C, 9395, 59003B, 59004B, 59003A, and 59004A. ARF was defined by ICD-9 518.81 or 518.84 or management codes 960, 9601, 9602, 9603, 9604, 9605, 9390, 9391, or 311. DM was identified by ICD-9 250. A patient was defined as having DM if the code appeared in ≥ 1 admission claim or ≥ 3 claims for ambulatory care. According to a previous study, the accuracy in identifying DM patients in Taiwan National Health Insurance Research Database using similar approaches was 92.4% with the code appearing ≥ 1 admission claim and 95.7% with the code appearing ≥ 4 claims for ambulatory care, respectively [25]. HBOT and ARF were included for the analysis of the association between severity of COP and risk for developing DM.

### Comparison of the risk for DM between the study and comparison cohorts

We compared the risk for DM between the study and comparison cohorts by following up until 2013 (Figure 2). We also performed a stratified analysis by age, sex, comorbidities, and follow-up periods to evaluate whether there was an effect modification from these variables. The follow-up periods were classified as <1 month, 1–6 months, 7–12 months, 1–2 years, 2–4 years, and ≥4 years, which were used for investigating the short- and long-term effects of COP on the development of DM.

### Ethics statement

The Institutional Review Board at Chi-Mei Medical Center approved this study, which was also strictly conducted according to the Declaration of Helsinki. Because the NPD and LHID2000 contain de-identified information of the participants, informed consent from the participants is waived, which does not affect the right and welfare of the participants.

### Data analysis

We used independent *t*-test to evaluate the differences in continuous variables and chi-square test to evaluate those in categorical variables in the comparison of demographic data, comorbidities, and monthly income between the study and comparison cohorts. In the comparison of the risk for DM between the two cohorts, we used Cox proportional hazard regression analysis with adjustment for age; sex; comorbidities of hypertension, hyperlipidemia, hyperuricemia, obstructive sleep apnea, coronary artery disease, congestive heart failure, hyperthyroidism, hypothyroidism, gestational DM, and polycystic ovary syndrome; and monthly income. In addition, we used Kaplan–Meier's method and log-rank test to compare the risk for DM between the two cohorts during the follow-up period. To identify the independent predictors for DM, we used Cox proportional hazard regression analysis first with a full model that included all variables studied and then a reduced model that included only the variables that were identified as significant predictors. In order to improve the precision, we also performed a propensity score matching by Cox proportional hazard regression analysis to investigate the independent predictors for DM. All the analyses were performed using SAS 9.4 for Windows (SAS Institute, Cary, NC, USA) at a two-tailed significance level of 0.05.

## CONCLUSIONS

This retrospective population-based cohort study showed that COP increased the risk for DM, especially in patients with a younger (<35 years) or older (≥65 years) age, female sex, and comorbidities of congestive heart failure, hyperthyroidism, and polycystic ovary syndrome. The effect of increased risk for DM developed immediately after COP and lasted for even more than 4 years. The disruption of the endocrine system including the brain and the pancreas due to the hypoxic injury and inflammatory and immunological reactions may play the role. Early follow-up of COP patients for the development of DM is recommended, especially for those who belong to the high-risk subgroups. Further intervention studies are warranted to validate the results and delineate the detailed mechanisms.

## References

[1] Buckley NA, Juurlink DN, Isbister G, Bennett MH, Lavonas EJ (2011). Hyperbaric oxygen for carbon monoxide poisoning. Cochrane Database Syst Rev.

[2] Ernst A, Zibrak JD (1998). Carbon monoxide poisoning. N Engl J Med.

[3] Pan YJ, Liao SC, Lee MB (2010). Suicide by charcoal burning in Taiwan, 1995–2006. J Affect Disord.

[4] Weaver LK (2009). Clinical practice. Carbon monoxide poisoning. N Engl J Med.

[5] Hampson NB, Hauff NM, Rudd RA (2009). Increased long-term mortality among survivors of acute carbon monoxide poisoning. Crit Care Med.

[6] Huang CC, Chung MH, Weng SF, Chien CC, Lin SJ, Lin HJ, Guo HR, Su SB, Hsu CC, Juan CW (2014). Long-term prognosis of patients with carbon monoxide poisoning: a nationwide cohort study. PLoS One.

[7] Zou JF, Guo Q, Shao H, Li B, Du Y, Liu M, Liu F, Dai L, Lin HJ, Su SB, Guo HR, Huang CC (2015). Lack of pupil reflex and loss of consciousness predict 30-day neurological sequelae in patients with carbon monoxide poisoning. PLoS One.

[8] Zou JF, Guo Q, Shao H, Li B, Du Y, Liu M, Liu F, Dai L, Chung MH, Lin HJ, Guo HR, Yang TM, Huang CC, Hsu CC (2014). A positive Babinski reflex predicts delayed neuropsychiatric sequelae in Chinese patients with carbon monoxide poisoning. Biomed Res Int.

[9] Thorens B (2011). Brain glucose sensing and neural regulation of insulin and glucagon secretion. Diabetes Obes Metab.

[10] Mounien L, Marty N, Tarussio D, Metref S, Genoux D, Preitner F, Foretz M, Thorens B (2010). Glut2-dependent glucose-sensing controls thermoregulation by enhancing the leptin sensitivity of NPY and POMC neurons. FASEB J.

[11] Marty N, Dallaporta M, Thorens B (2007). Brain glucose sensing, counterregulation, and energy homeostasis. Physiology (Bethesda).

[12] Mobbs CV, Isoda F, Makimura H, Mastaitis J, Mizuno T, Shu IW, Yen K, Yang XJ (2005). Impaired glucose signaling as a cause of obesity and the metabolic syndrome: the glucoadipostatic hypothesis. Physiol Behav.

[13] Thorens B (2008). Glucose sensing and the pathogenesis of obesity and type 2 diabetes. Int J Obes (Lond).

[14] Thorens B (2010). Central control of glucose homeostasis: the brain--endocrine pancreas axis. Diabetes Metab.

[15] Seeley RJ, Sandoval DA (2016). Targeting the brain as a cure for type 2 diabetes. Nat Med.

[16] De Flines J, Scheen AJ (2007). [Congestive heart failure and diabetes mellitus: an intricated relationship]. [Article in French]. Rev Med Liege.

[17] Wang C (2013). The relationship between type 2 diabetes mellitus and related thyroid diseases. J Diabetes Res.

[18] Orio F, Muscogiuri G, Nese C, Palomba S, Savastano S, Tafuri D, Colarieti G, La Sala G, Colao A, Yildiz BO (2016). Obesity, type 2 diabetes mellitus and cardiovascular disease risk: an uptodate in the management of polycystic ovary syndrome. Eur J Obstet Gynecol Reprod Biol.

[19] Legro RS, Kunselman AR, Dodson WC, Dunaif A (1999). Prevalence and predictors of risk for type 2 diabetes mellitus and impaired glucose tolerance in polycystic ovary syndrome: a prospective, controlled study in 254 affected women. J Clin Endocrinol Metab.

[20] Biessels GJ, Kappelle LJ (2012). The treatment of diabetes after an acute ischaemic stroke. Eur Neurol Rev.

[21] Uyttenboogaart M, Koch MW, Stewart RE, Vroomen PC, Luijckx GJ, De Keyser J (2007). Moderate hyperglycaemia is associated with favourable outcome in acute lacunar stroke. Brain.

[22] Luitse MJ, Biessels GJ, Rutten GE, Kappelle LJ (2012). Diabetes, hyperglycaemia, and acute ischaemic stroke. Lancet Neurol.

[23] National Health Insurance Administration (2014). National Health Insurance Annual Report 2014–2015.

[24] Lee CC, Lee MT, Chen YS, Lee SH, Chen YS, Chen SC, Chang SC (2015). Risk of aortic dissection and aortic aneurysm in patients taking oral fluoroquinolone. JAMA Intern Med.

[25] Lin CC, Lai MS, Syu CY, Chang SC, Tseng FY (2005). Accuracy of diabetes diagnosis in health insurance claims data in Taiwan. J Formos Med Assoc.

